# Widespread introduced species dominate the urban tree assemblage on the endemic‐rich tropical island of São Tomé

**DOI:** 10.1002/ece3.70153

**Published:** 2024-09-01

**Authors:** Lena Strauß, Ricardo F. de Lima, Timothy R. Baker, Laura Benitez Bosco, Gilles Dauby, Olivier Lachenaud, Angela Lima, Dilson Madre Deus, Maria do Céu Madureira, Estevão Soares, Pascoal Sousa, Tariq Stévart, Martin Dallimer

**Affiliations:** ^1^ Sustainability Research Institute, School of Earth and Environment University of Leeds Leeds UK; ^2^ cE3c – Centre for Ecology, Evolution and Environmental Changes University of Lisbon Lisbon Portugal; ^3^ CHANGE – Global Change and Sustainability Institute University of Lisbon Lisbon Portugal; ^4^ Departamento de Biologia Animal, Faculty of Sciences University of Lisbon Lisbon Portugal; ^5^ Gulf of Guinea Biodiversity Center São Tomé São Tomé and Príncipe; ^6^ School of Geography University of Leeds Leeds UK; ^7^ Fauna & Flora Cambridge UK; ^8^ Fundação Príncipe Santo António São Tomé and Príncipe; ^9^ CIBIO – Centro de Investigação em Biodiversidade e Recursos Genéticos, InBIO Laboratório Associado, Campus de Vairão Universidade do Porto Vairão Portugal; ^10^ BIOPOLIS – Program in Genomics, Biodiversity and Land Planning CIBIO, Campus de Vairão Vairão Portugal; ^11^ AMAP – botAnique et Modélisation de l'Architecture des Plantes et des végétations Université Montpellier, CIRAD, CNRS, INRAE, IRD Montpellier France; ^12^ Meise Botanic Garden Meise Belgium; ^13^ Herbarium et Bibliothèque de Botanique africaine Université Libre de Bruxelles Brussels Belgium; ^14^ Direcção das Florestas e da Biodiversidade São Tomé São Tomé and Príncipe; ^15^ Ministério da Educação, Cultura e Ciências São Tomé São Tomé and Príncipe; ^16^ Departamento de Ciências da Vida, Centre for Functional Ecology Universidade de Coimbra Coimbra Portugal; ^17^ Associação Monte Pico Monte Café São Tomé and Príncipe; ^18^ Parque Natural do Obô de São Tomé Bom Sucesso São Tomé and Príncipe; ^19^ Africa and Madagascar Department Missouri Botanical Garden St. Louis Missouri USA; ^20^ Centre for Environmental Policy Imperial College London London UK

**Keywords:** Afrotropical forest, anthropogenic gradient, NMDS, oceanic island, urban ecology, urbanisation

## Abstract

The Afrotropics are experiencing some of the fastest urbanisation rates on the planet but the impact of city growth on their rich and unique biodiversity remains understudied, especially compared to natural baselines. Little is also known about how introduced species influence β‐diversity in these contexts, and how patterns coincide with native ranges of species. Here we investigated how tree assemblages of the endemic‐rich Afrotropical island of São Tomé differed between urban, rural and natural zones. These were primarily characterised by urban greenspaces, shade plantations, and old‐growth forests, respectively. Based on 81 transects, we assessed biodiversity metrics of endemic, native and introduced species. Tree abundance and species richness were highest in the natural zone, where the composition was most different from the urban zone. The tree community of the rural zone was the most uneven and had the least variation among transects, representing the lowest β‐diversity. The urban zone was dominated by introduced species (57.7%), while the natural zone hosted almost exclusively native species (93.3%), including many endemics (26.1%). The biogeographic realms that species originated from were particularly diverse in the urban zone, with few species from the Afrotropics. In contrast to native and endemic trees, introduced trees were clearly associated with urban and rural expansion, as they were much more abundant and species‐rich in these zones than in the natural zone, facilitating biotic homogenisation. These findings highlight how urban and rural environments are affecting the native tree flora of São Tomé, and the need for conservation measures geared towards globally threatened and endemic tree species. Importantly, these require the protection of natural forests, despite the rising land demands for settlements and agriculture. Ultimately, such action to conserve endemic trees will contribute to global efforts to prevent further biodiversity declines.

## Graphical abstract

Afrotropical cities are growing rapidly but how this impacts biodiversity is largely unclear. Our study on São Tomé Island, Central Africa, reveals that the urban tree assemblage is impoverished and dominated by introduced species, while the natural forest is essential for the protection of native and endemic species.

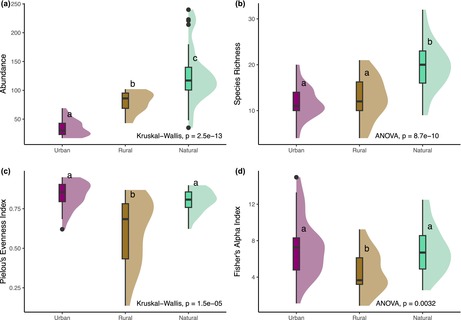

## 1 INTRODUCTION

By 2100, urban areas around the globe will occupy approximately six times the area they covered in 2000 (Gao & O'Neill, 2020). Urban expansion, especially into relatively intact areas, can have substantial impacts on biodiversity, but our understanding of these effects is dominated by studies concentrating on biotic or abiotic factors, city age or size, and management practices within cities themselves (Beninde et al., 2015). Research that extends beyond city boundaries also typically only reaches nearby rural hinterlands (Rega‐Brodsky et al., 2022), which often support ecological communities that are already highly influenced by human interference. We rarely compare species assemblages in urban areas against those of natural or near‐natural zones (Padilla & Sutherland, 2019). Thus, our understanding of the extent to which urbanisation impacts biodiversity is compromised.

The lack of natural or near‐natural baselines helps to explain why assessments of urban biodiversity come to surprisingly different conclusions (McKinney, 2008). Focussing on α‐diversity, some studies have found reduced diversity, while others report that urban areas can be hotspots of plant diversity (Gillespie et al., 2017; Kantsa et al., 2013). Often, high species richness in urban environments has been linked to increased habitat heterogeneity, i.e. the occurrence of many ecological niches due to a variety of mosaic patches, and to the deliberate or unintentional introduction of non‐native species by humans (Kowarik, 2011). Some cities in Australia have not only been shown to be more diverse in plants, however, but also to outnumber their rural surroundings in rare and threatened species (Ives et al., 2016), including the urban‐restricted tree species *Grevillea caleyi* (Soanes & Lentini, 2019). One explanation to this could be that pre‐existing biodiversity hotspots may have persisted in urban areas (Spotswood et al., 2021).

A more complete understanding of biodiversity changes, especially in the context of disturbed sites, usually requires the analysis of β‐diversity; however, this is often neglected (Mori et al., 2018). By examining both α‐ and β‐diversity, it becomes clearer if species richness in individual land‐use types accompanies shifts in species compositions across land‐use types. Such turnovers can be driven by introduced species, and thus the study of biogeographic origins may reveal important insights into underlying mechanisms. The proliferation of widespread introduced species at the expense of native species, for instance, may cause biotic homogenisation. As an important facet of the current biodiversity crisis, this ecological process describes taxonomic, genetic, or functional assimilation in two or more localities (β‐diversity) over time, resulting from an imbalance in species introductions and extinctions (Olden et al., 2016). Biotic homogenisation has been strongly connected with land‐use change (Kramer, Zwiener, & Müller, 2023), and with urban expansion in particular (Lokatis & Jeschke, 2022). Exploring homogenising processes may hence advance our comprehension of urbanisation‐induced biodiversity changes.

By looking at native ranges of species, we also get a clearer picture of the risk of regional biotic homogenisation. The further away the native biogeographic realm, the further species have travelled and the more widespread they may become as a result. Some may even originate from multiple realms, and are likely associated with generalist traits, thus being highly adaptable to anthropogenic disturbance (Kramer, Bald, et al., 2023). Nonetheless, few studies have dealt with urbanisation and native ranges (Hunte et al., 2019), particularly from the perspective of β‐diversity.

It is important to study the impact of urban growth in the tropics, where this land‐use change is happening very rapidly. The Afrotropics are particularly relevant in this regard as they have some of the world's fastest urbanisation rates (OECD/SWAC, 2020). Afrotropical cities are often surrounded by agroforestry systems (Zomer et al., 2016), in which trees are key structural elements that support other taxa, including forest‐dependent species (Deheuvels et al., 2014). These rural surroundings may therefore host high levels of biodiversity, which makes them unlike many counterparts in temperate zones that are typically mono‐structured agricultural systems with limited tree cover. Accordingly, we cannot expect that urban biodiversity patterns in tropical cities replicate those of temperate zones, where tree diversity may be higher in cities than in rural surroundings.

To assess the impacts of urbanisation on biodiversity, oceanic islands are ideal study systems because they are characterised by a high proportion of range‐restricted species that are often sensitive to anthropogenic changes (Whittaker et al., 2023). However, they are rarely considered in urban biodiversity research (Lowry et al., 2020). High extinction and introduction rates on islands have also led to biotic homogenisation (Castro et al., 2010), which may further exacerbate patterns linked to urbanisation. While the importance of tree and canopy cover in urban areas for delivering ecosystems services is relatively well studied, even in the tropics, we still know little about how tree species assemblages are being altered by urbanisation. Here we investigated how tree communities differed between urban, rural and natural zones on the oceanic island of São Tomé, Central Africa, by answering the following questions:

1. How do land‐use types, i.e. urban, rural and natural zones, influence tree community composition?
2. To what extent do introduced species drive these differences?
3. How are native biogeographic ranges of tree species distributed across zones?

We hypothesised that rural zones, due to the presence of agroforests, would be considerably richer in tree diversity than urban zones, but that natural zones would be most diverse (Deheuvels et al., 2014). In addition, we expected a strong impact of widespread introduced species on tree community patterns in human‐modified environments (de Lima et al., 2014), and an overall dominance of Afrotropical species across zones.

## 2 MATERIALS AND METHODS

### 2.1 Study area

**FIGURE 1:**
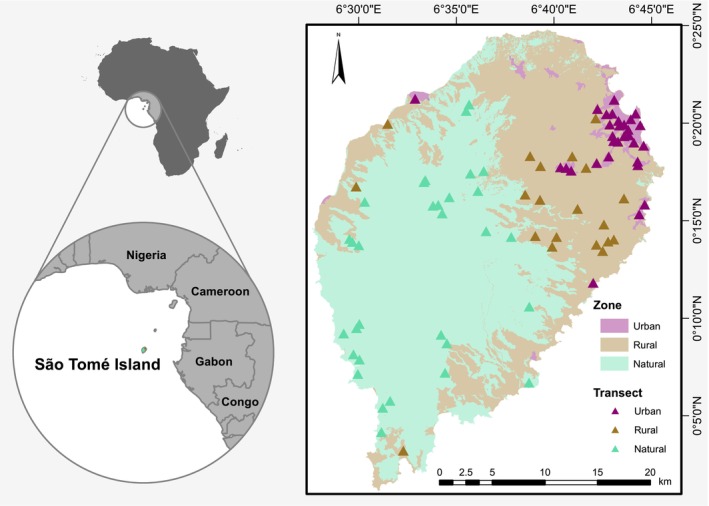
Location of São Tomé Island in Africa (top left) and in the Gulf of Guinea (bottom left), and of the 81 transects sampled across urban, rural and natural zones of São Tomé (right). Zonation of the latter was based on Soares et al. (2020) and Ministério das Infra‐Estruturas, Recursos Naturais e Ambiente (2018).

São Tomé is an oceanic island of 857 km^2^ that, together with the smaller Príncipe, comprises the Democratic Republic of São Tomé and Príncipe. It is part of the Guinean Forests of West Africa Biodiversity Hotspot, hosting an endemic‐rich but threatened biodiversity (de Lima et al., 2022). Isolated in the Gulf of Guinea, the country is a distinct bioregion, whose vascular flora is composed of approximately 14.5% endemic species (Stévart et al., 2022), and is the best studied in the Afrotropics (Droissart et al., 2018). The climate is oceanic equatorial (mean annual temperatures 16.2–25.9°C; mean annual precipitation 600–7000 mm) with one main dry season between June and August, and a shorter one between December and January. Due to the mountainous centre and prevailing winds from the southwest, the island has a distinct rain shadow (Ceríaco et al., 2022). There is also a strong gradient of human impacts linked to ruggedness (Norder et al., 2020). Lowland forests have been largely converted to agriculture, while most remaining forest is found in the mountainous centre (Dauby et al., 2022). Urban areas, where around three quarters of the population reside, are mostly located near the coast in the drier northeast (Figure 1). As in other African countries, the population, economy, and politics are disproportionately centred around the capital (Güneralp et al., 2018). The urban population has increased five‐fold since 1950; a rate that far exceeds that of Central Africa as a whole (UN‐DESA, 2018).

### 2.2 Sampling design

#### 2.2.1 Land classification

To assess the effect of urbanisation, we classified São Tomé into urban, rural and natural zones, based on land‐use (Soares et al., 2020) and urban area maps (Ministério das Infra‐Estruturas, Recursos Naturais e Ambiente, 2018). The urban zone, covering 24 km^2^ (2.8%) of the island, included greenspaces such as public parks, home gardens, and secondary forest fragments, besides infrastructure and sealed surfaces. The rural zone of 345 km^2^ (40.3%) comprised landscapes of low human population density with forested or non‐forested plantations and areas of regenerating vegetation. This zone predominantly corresponded to shade plantations, an agroforestry system composed of tall, typically planted trees that shade understorey cash crops such as coffee (*Coffea* spp.) or cocoa (*Theobroma cacao*). The natural zone of 488 km^2^ (56.9%), most of which (252 km^2^) is protected as Obô Natural Park, spanned old‐growth native and secondary forests with limited human presence. Illegal practices including selective logging, hunting, and charcoal production have however been taking place within and beyond the natural zone over decades, largely because of poor law enforcement (de Lima et al., 2022). Nonetheless, the natural zone contains the best‐preserved forests of the island, which have qualified as Global 200 Ecoregion (Olson & Dinerstein, 2002).

#### 2.2.2 Sampling strategy

We established 81 transects across the three zones. For the natural zone, 31 transects were purposefully selected to capture the diversity of well‐preserved forest across altitudinal and rainfall gradients. For urban and rural zones, we used a stratified random sampling approach. First, we created a 500 × 500 m grid across the island and selected 30 grid cells in the urban zone and 20 in the rural zone. Fewer rural grid cells were selected as this zone is more uniform (de Lima et al., 2014). A transect was then established in each grid cell. If this was not possible, we targeted a new randomly selected grid cell. Across all zones, each transect consisted of four 5 × 50 m sections (0.1 ha) separated by less than 50 m, ensuring at least 200 m between transects. Sections were not in a straight line so that a homogenous patch of habitat could be sampled by avoiding obstacles such as roads or rivers/valleys (Benitez Bosco et al., 2018).

### 2.3 Data collection

#### 2.3.1 Tree sampling

Fieldwork was carried out from October 2019 to August 2021. All trees with a diameter at breast height ≥ 5 cm were identified within each transect section (Benitez Bosco et al., 2018). Plants that were monocots, namely from the Arecaceae (e.g. *Elaeis guineensis*, *Cocos nucifera*), Caricaceae (*Carica papaya*), Musaceae (*Musa* spp.), Pandanaceae (*Pandanus thomensis*), and Poaceae (*Bambusa vulgaris*), were not considered to be trees and thus excluded. These were likely to respond differently to land‐use change compared to dicots (Renninger & Phillips, 2016). We photographed and collected every species at least once to facilitate identification down to the lowest taxonomic level (Tropicos, 2023).

#### 2.3.2 Species origins

We distinguished introduced and native taxa, classifying the latter as endemic if the native distribution was restricted to the oceanic islands of the Gulf of Guinea (Table A1). We ascribed each taxon to a biogeographic realm (Udvardy, 1975), but if data were unavailable, no realm was ascribed. All information was based on POWO (2023) and Figueiredo et al. (2011), or on author expertise if information was incomplete or doubtful.

#### 2.3.3 Environmental parameters

We selected spatially explicit environmental variables that best reflected physico‐climatic gradients, based on availability and relevance, to explain differences in tree assemblages (Dauby et al., 2022; Soares et al., 2020). The resolution of altitude, precipitation, remoteness, slope, and topography was ~90 m (Soares et al., 2020), while for cloud cover it was 1 km (Wilson & Jetz, 2016). All variables were continuous, except for topography, which was categorical, distinguishing flat areas, valleys, middle slopes, upper slopes, and ridges. For each of the continuous variables, we used the mean of the values extracted at start and end points of transect sections to characterise each transect. For topography, we used the most frequent category among these start and end points, and for those eight transects where categories were equally frequent, we used the category that occurred first along the transect.

### 2.4 Data analyses

Analyses were performed in R 4.2.2 (R Core Team, 2022), for which we created a community data matrix, consisting of abundance per species (columns) and transect (rows). To test for differences in α‐diversity between zones, we calculated abundance and species richness on transect level from this data, as well as evenness, which accounts for the abundance of each species relative to the abundances of other species, and Fisher's alpha to describe the relationships between abundance and species richness. For species richness and Fisher's alpha index, we used one‐way ANOVAs followed by Tukey HSD post‐hoc tests since data was normally distributed and homoscedastic. For abundance and Pielou's evenness index, we employed Kruskal‐Wallis rank sum tests followed by pairwise Wilcoxon rank sum tests.

Using “BiodiversityR” (Kindt & Coe, 2005), we created rank‐abundance curves and Rényi diversity profiles, that both combine the measures of species richness and evenness, and compare them across zones (Oldeland et al., 2010). Rank‐abundance curves sort species by their abundance. A completely horizontal curve would determine perfect evenness among species, which is also true for Rényi diversity profiles. As a type of diversity ordering technique, a Rényi diversity profile orders the most common diversity indices ranging between richness and evenness, but it lacks information on the proportions of species. It does however enable straightforward comparisons between measures such as Shannon and Simpson diversity indices. This allows for a more comprehensive picture than a single diversity index. The highest curve indicates the highest diversity among zones, but if curves intersect, this inference cannot be made (Kindt & Coe, 2005; Oldeland et al., 2010). To account for different sample sizes, we calculated average abundance and species richness per transect for each zone. In addition, we created sample‐based species accumulation curves to evaluate the variation in expected mean species richness between zones. We further used Chao, first‐ and second‐order Jackknife, and Bootstrap to extrapolate curves to estimate total species richness per zone.

To examine whether species origins affected patterns across zones, we tested for differences in abundance and species richness for native and introduced species, and for endemic‐ and non‐endemic species. This was done via one‐way ANOVAs followed by Tukey HSD post‐hoc tests when assumptions were met, or otherwise by Kruskal‐Wallis rank sum tests followed by pairwise Wilcoxon rank sum tests.

We calculated indicator values to identify associations between species and zones, which are maximised when a species occurs on all transects (high fidelity) of one zone (high specificity) (Dufrene & Legendre, 1997). To this end, we used the multi‐level pattern analysis of the function “multipatt” from “indicspecies” (de Cáceres & Legendre, 2009), and the argument “func = “IndVal.g”” for unequal group sizes. This function accounts for different niche breadths of species by exploring both individual zones and combinations.

To analyse variability of tree communities between zones (β‐diversity), we first standardised community data applying the highly robust “hellinger” method from the function “decostand” in “vegan” (Oksanen et al., 2022). We then used non‐metric multidimensional scaling (NMDS) to visualise the differences in the structure of tree assemblages between zones. We deemed stress values below 0.2 to be acceptable (Clarke, 1993).

To help interpret the NMDS, we tested differences in location through permutational multivariate analysis of variance (PERMANOVA; permutations = 999) by using “adonis2” in “vegan” and “pairwise.adonis2” in “pairwiseAdonis” (Martinez Arbizu, 2017), and in dispersion through permutational multivariate analysis of dispersion (PERMDISP; permutations = 999) by using “betadisper” and “permutest.betadisper” in “vegan”. Location refers to the centroid of all transects within a zone and dispersion refers to the variation among transects within a zone. This was complemented by an analysis of similarities (ANOSIM; permutations = 999) via “anosim” in “vegan”, in which ranked dissimilarities between transects are used to test whether differences are greater within or between zones.

We also generated two NMDS plots based on species scores to visualise biogeographic origins and realms (native ranges), respectively, as drivers of tree assemblage structures. To understand how tree composition was linked to environmental characteristics, we superimposed all significant environmental variables as arrows on the NMDS, standardising continuous environmental variables using “standardise” from the function “decostand” in “vegan”.

In addition, we aimed to quantify the extent to which zone (urban, rural, natural) and spatial structures as described through principal coordinates of neighbour matrices (PCNM) influence tree assemblages, alongside the abovementioned environmental variables (altitude, precipitation, remoteness, slope, topography, cloud cover). For this purpose, we employed variation and hierarchical partitioning via “rdacca.hp”, which are two complementary methods that do not limit the number of predictors and thus can avoid some of the errors associated with selection procedures in regression models (Lai et al., 2022). The former determined the unique and average shared contributions and the latter the overall importance of each predictor (or group of predictors), namely PCNM (Borcard & Legendre, 2002), environment, and zone, towards explained variation (*R*‐squared) in tree assemblages.

## 3 RESULTS

We removed 21 individuals (0.32%) only determined to family or higher, and 287 individuals (4.37%) that were identified to genus, but for which we could not exclude the possibility that they may belong to already identified species. The final dataset contained 6563 individuals, 6436 belonging to 171 species, and 127 to undetermined species within 6 genera. For simplicity, all are referred to as species in the remainder of this work. There were between 4 and 32 species and between 17 and 240 individuals per transect. The mean number of species and individuals per transect was 15.0 (±5.7) and 81.0 (±51.6), respectively. The population density for the urban zone was 348.0 individuals/ha, for the rural zone 795.0 individuals/ha, and for the natural zone 1267.4 individuals/ha.

**FIGURE 2:**
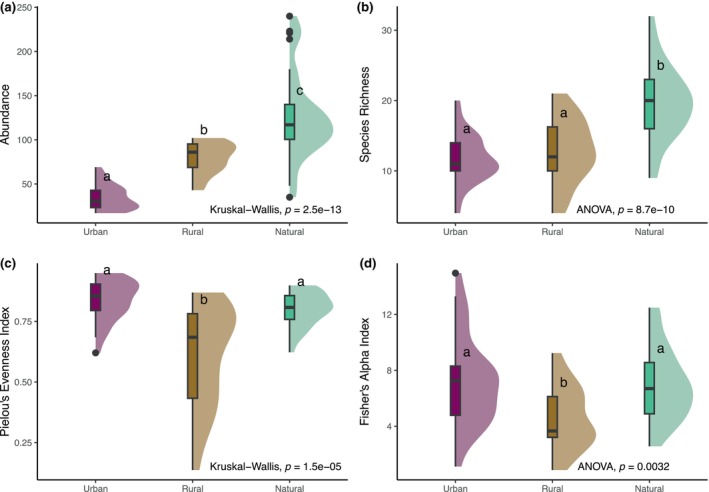
Violin box plots of (a) abundance, (b) species richness, (c) Pielou's evenness index, and (d) Fisher's alpha index per transect, showing the significance of relationships between zones. *p*‐Values (df = 2) of one‐way ANOVA, (b) *F* = 27.590 and (d) *F* = 6.192, or Kruskal–Wallis rank sum tests, (a) *χ* ^2^ = 58.067 and (c) *χ* ^2^ = 22.185, at the bottom of each graph. Significant differences from Tukey HSD or pairwise Wilcoxon rank sum tests indicated by different superscript letters. Maximum width of violins scaled to 1.

**FIGURE 3:**
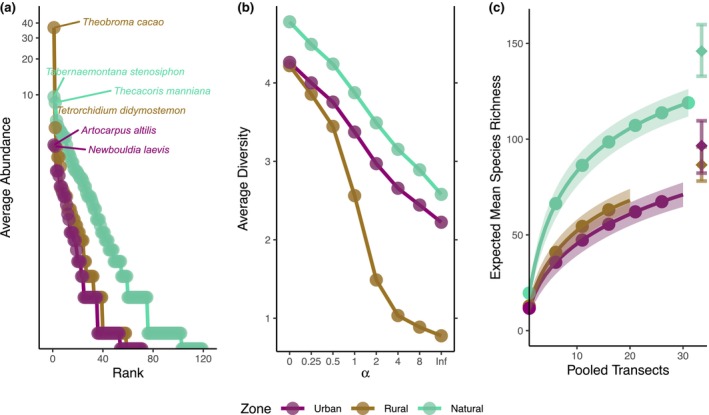
Curves grouped according to zones. (a) Rank‐abundance curves on logarithmic scale, displaying the names of the two most abundant tree species for each zone. (b) Rényi diversity profiles, where the value of alpha stretches from zero to infinity (ranging between richness and evenness). 0: ln(richness); 1: Shannon; 2: ln(1/Simpson); Inf: Ln(1/BergerParker), which is the dominance of the most abundant tree species. For (a) and (b) abundance and diversity were averaged per transect for each zone, respectively. (c) Sample‐based species accumulation curves with 95% confidence intervals. The vertical bars indicate the interval between the minimum (Bootstrap) and maximum species richness estimator (second order Jackknife) per zone, while the diamond shapes show the respective mean.

Abundance (Figure 2a) and species richness (Figure 2b) were highest in the natural zone (α‐diversity). Abundance was higher in rural than in urban zones, but there was no difference in species richness between the two. For Pielou's evenness (Figure 2c) and Fisher's alpha indices (Figure 2d), transects in the rural zone had the lowest values compared to urban and natural zones, which were similar among each other. The hyperdominance of cocoa made the rural zone the steepest and thus most uneven rank‐abundance curve (Figure 3a). The natural zone had the most diverse profile, and the rural zone was the least diverse (Figure 3b). Species richness accumulated more rapidly and was highest in the natural zone (119 species) compared to the rural and urban zones, even though it was incompletely assessed in all zones (Figure 3c).

**FIGURE A1:**
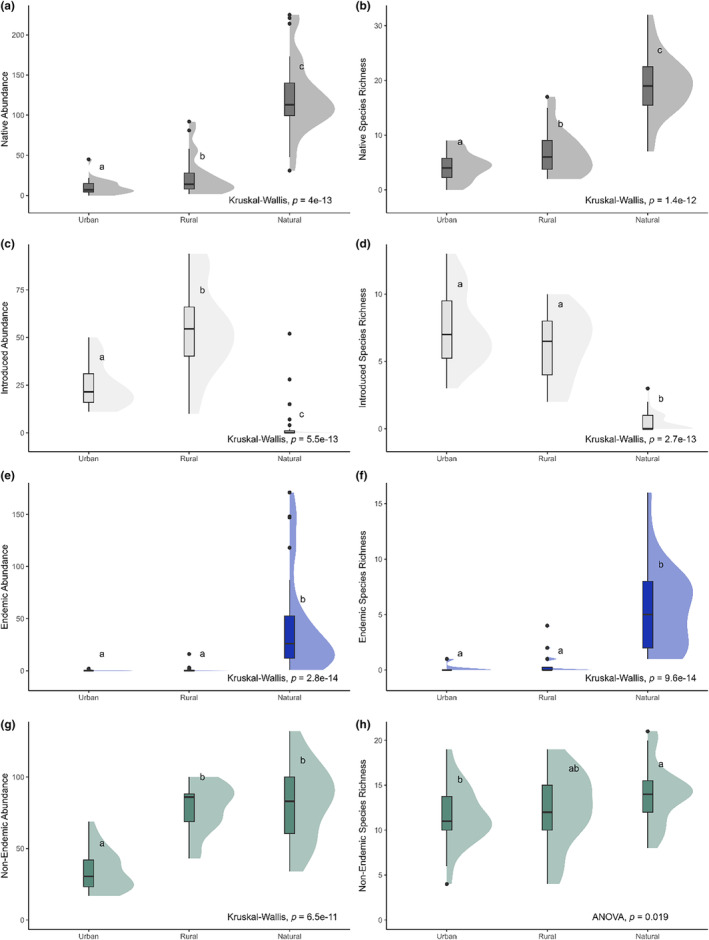
Violin box plots of tree abundance and species richness of native (a and b), introduced (c and d), endemic (e and f), and non‐endemic taxa (g and h) per transect, showing the significance of relationships between zones. *p*‐Values (df = 2) of one‐way ANOVA, (h) *F* = 4.147, or Kruskal‐Wallis rank sum tests, (a) *χ* ^2^ = 57.111, (b) *χ* ^2^ = 54.572, (c) *χ* ^2^ = 56.473, (d) *χ* ^2^ = 57.904, (e) *χ* ^2^ = 62.395, (f) *χ* ^2^ = 59.957, and (g) *χ* ^2^ = 46.902, at the bottom of each graph. Significant differences from Tukey HSD or pairwise Wilcoxon rank sum tests indicated by different superscript letters. Maximum width of violins scaled to 1.

The abundance and richness of native and endemic species were significantly higher in the natural zone than in the rural and urban zones (Figure A1). Contrastingly, the abundance and richness of introduced species was significantly higher in both the urban and rural zones than in the natural zone. In the urban zone, introduced species accounted for 70.6% of the abundance and 57.7% of the richness, contrasting to 68.3% and 36.8% in the rural zone, and 2.9% and 6.7% in the natural zone, respectively. Endemics in the natural zone accounted for 34.5% of the abundance and 26.1% of the richness, contrasting to 1.4% and 7.4% in the rural zone, and 0.6% and 1.4% in the urban zone.

We identified 71 indicator species. The top urban zone indicator species were introduced from Indomalaya (mango, *Mangifera indica*) and native Afrotropical (boundary tree, *Newbouldia laevis*), while those of the rural zone were introduced from the Neotropics (cocoa and coral tree, *Erythrina poeppigiana*; Table A2). The top indicator species for rural and urban zones combined were from Oceania (breadfruit, *Artocarpus altilis*) and Indomalaya (jackfruit, *Artocarpus heterophyllus*). In contrast, the main natural zone indicator species were native Afrotropical species (*Homalium henriquesii* and *Casearia barteri*).

**FIGURE 4:**
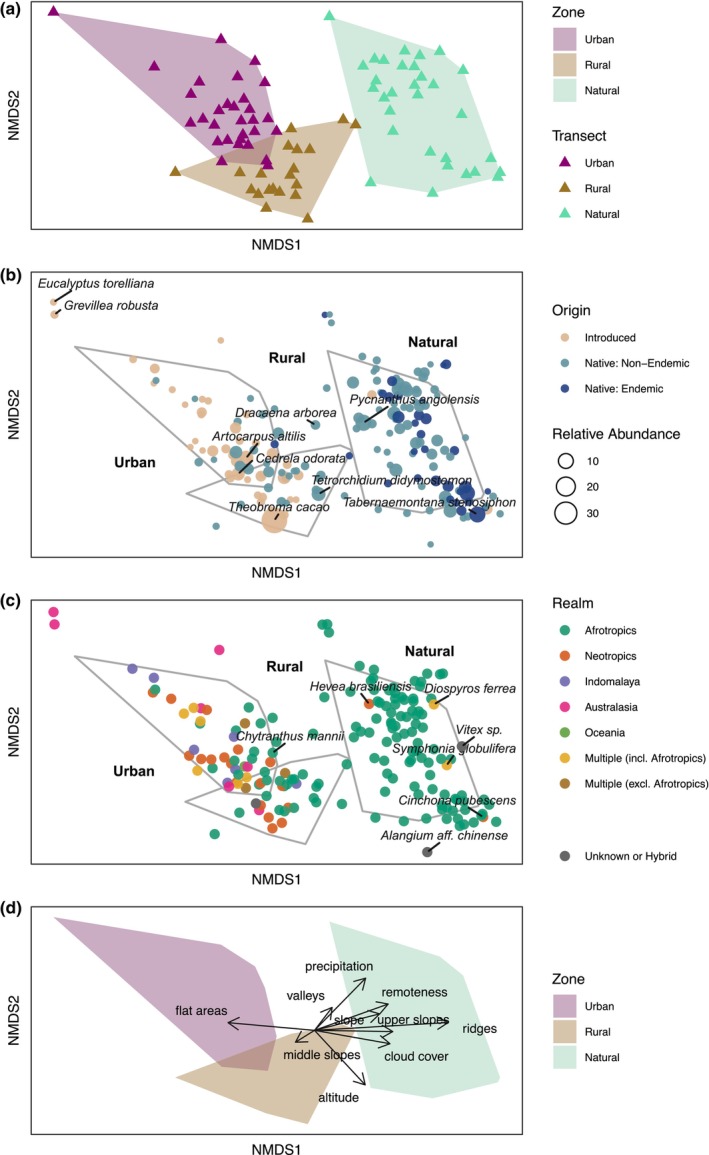
Non‐metric multidimensional scaling (NMDS) plots based on Bray‐Curtis dissimilarities of tree species abundances along transects (stress value = 0.126). (a) Structural differences between transects. (b) Structural differences between species, differentiated by origin and abundance relative to sample size of zones. The names of the two most abundant species of each zone and of those in outstanding positions are displayed: *Dracaena arborea* was a significant indicator for urban and natural transects combined (Table A2); *Eucalyptus torelliana* and *Grevillea robusta* were only recorded in one transect in a tree nursery of the capital. (c) Structural differences between species, differentiated by realm (native range). The names of species with non‐Afrotropical native ranges that are associated with the natural zone are displayed, as well as endemic species that are associated with non‐natural zones. (d) Structural differences between transects, with fitted vectors of six environmental variables (*R* ^2^ = .489 to .698, *p* = .001). In (a) and (d) minimum convex hulls for each zone are shown. In (b) and (c), these hulls are indicated by lines, and some minor random variation (“jitter”) has been added to avoid overlap between points (species).

**FIGURE A2:**
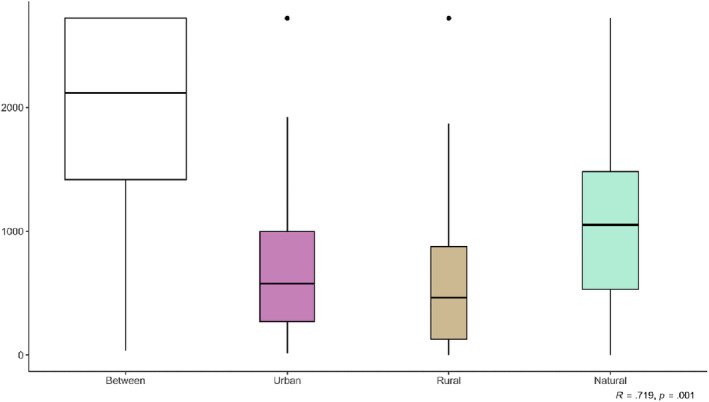
Analysis of similarities in tree assemblages based on dissimilarity ranks (*y*‐axis) between zones (“Between”) and within zones (“Urban”, “Rural”, “Natural”), with associated *R*‐ and *p*‐value. The *R*‐value can range between 1 (high dissimilarities between zones) and close to 0 (equal dissimilarities between and within zones).

Regarding β‐diversity, tree assemblages were distinct between all zones, but most notably between natural and urban zones (pairwise PERMANOVA: *F* = 21.369, *p* = .001; Figure 4a). Floristic similarity was always lower between zones than within zones (ANOSIM: *R* = .719, *p* = .001). Within zones, tree assemblages were equally similar among urban and rural transects (pairwise PERMDISP: *F* = 6.019, *p* = .235; Figure A2), and significantly more distinct among natural transects (*p* < .050).

Biogeographic origins differed across zones, with many native and particularly endemic species only being present in the natural zone (Figure 4b). Of all species, 72.3% were exclusively Afrotropical and found across zones, even though many were more abundant in the natural zone. In contrast, the urban zone included the complete range of origins, i.e. many species were introduced from the Neotropics, Indomalaya, Australasia, Oceania, or multiple realms (Figure 4c).

All six environmental variables yielded significant correlations with the NMDS axes (Figure 4d). The first axis was strongly and positively associated with cloud cover, remoteness, slope, ridges, and upper slopes, and negatively with flat areas, while the second axis had weak positive associations with precipitation and weak negative ones with altitude. Additionally, the natural zone was associated to ridges, upper slopes, higher altitude and precipitation, while the urban zone was linked to flat areas and lower altitude, and the rural zone to middle slopes and lower precipitation.

**FIGURE A3:**
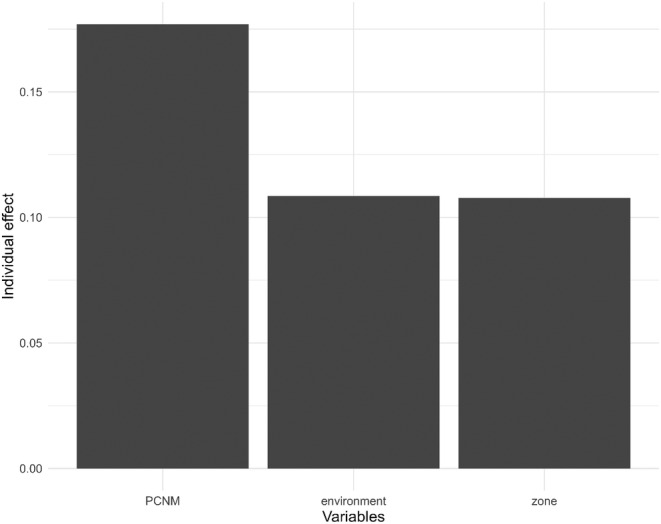
Hierarchical partitioning, combining unique and common fractions, of PCNM (principal coordinates of neighbour matrices), environment (altitude, precipitation, remoteness, slope, topography, cloud cover), and zone (urban, rural, natural) towards explained variation (*R*‐squared) of tree assemblages.

While 60.7% of the variation in tree assemblages remained unexplained, 17.7% was attributable to spatial factors (PCNM), 10.9% to environment, and 10.8% to zone (Figure A3). Within environment, precipitation and altitude were the most important variables (Table A3). According to variation partitioning, PCNM had the highest unique fraction (8.4%) and contributed to the highest shared fractions of the variation (Table A4).

## 4 DISCUSSION

On the tropical oceanic island of São Tomé, urban and rural tree species richness were similar, but tree assemblages in both zones were less diverse than that of the natural zone. This was particularly noticeable when considering the abundance and richness of native and endemic species. Introduced species, in contrast, abounded in urban and rural zones, implying that tree assemblages in human‐dominated landscapes have been subject to biotic homogenisation.

### 4.1 How do land‐use types influence tree community composition?

The highest tree abundance and species richness were found in the natural zone. This finding differs from some of the findings from biodiversity studies that consider other taxonomic groups on São Tomé, namely birds (Soares et al., 2020) and land snails (Tavares, 2021). These tended to have higher abundances and more species in land‐use types with higher anthropogenic influence. This is not surprising since the response of biodiversity to anthropogenic interference is taxa‐dependent (Barlow et al., 2007). The diversity patterns of tree species also contrast with the high plant diversity described for many temperate urban areas (Gillespie et al., 2017; Kantsa et al., 2013). One factor might be city age since the tropics tend to have younger cities (Aronson et al., 2014), in which plant communities may have had less time to adapt to urbanisation processes. The combined effect of higher natural tree diversity (Mittelbach et al., 2007) and less impoverished baselines in tropical compared to temperate areas (Wania et al., 2006) could further explain this discrepancy, highlighting the importance of studying biodiversity beyond urban and rural boundaries.

The lowest evenness and Fisher's alpha were found in the rural zone, which is dominated by shade plantations, an agroforestry system that is heavily managed to produce a few crops shaded by a small subset of fast‐growing species (Dauby et al., 2022). The rural tree assemblage of São Tomé might be particularly impoverished and uniform due to the history of intensive land‐use change, which contrasts to many countries in continental Africa, where cocoa production, for instance, depended on smallholdings instead of industrial‐scale plantations (Frynas et al., 2003).

Even though 16 species were shared across all zones, the assemblage structure was distinct between zones, especially between urban and natural zones (Figure 4a). It could be argued that these differences in tree compositions are a product of biophysical and climatic rather than anthropogenic factors. We observed that zone, space, and environmental variables were not independent and that tree assemblages strongly correlated with precipitation and altitudinal gradients. Microhabitat conditions, which may not be captured by the scale that environmental variables were assessed at, may further explain differences in species compositions. In addition, intra‐ or interspecific interactions could be influential.

Originally, São Tomé was almost entirely forested, but humans heavily altered these ecosystems. While native vegetation persisted in rugged areas at higher elevations (Norder et al., 2020), rural and urban zones were established in more accessible, drier areas at lower altitudes. Some of our natural transects were located at lower elevation but did not form any clusters in the NMDS (Figure 4a). This indicates that tree assemblages in the lowlands may have previously not been that different from those in higher elevations. Hence, the absence of many native species from rural and urban zones may be due to human actions rather than being driven by biophysical and climatic factors. Trees in these zones tend to be managed, being planted or removed to satisfy well‐defined human needs (de Lima et al., 2014). Nonetheless, some uncertainty remains with regard to whether some native species might have more specialist habitat requirements and would, therefore, have been absent without any human interference. This contrasts with the presence of introduced species in rural and urban zones, which is clearly attributable to anthropogenic factors. Besides, recent rapid urban expansion into rural areas (Muñoz‐Torrent et al., 2022) may help explain some floristic overlap between these two zones.

### 4.2 To what extent do introduced species drive these differences?

Native tree species, and notably the endemics, were clearly associated with the natural zone, contrasting to the anthropogenic affinities of introduced taxa. Biotic homogenisation was noticeable in our study area, since the occurrence of introduced species appeared to coincide with a drastic reduction in the number of native species. Homogenising processes were seemingly connected with land‐use change (Sánchez‐Ortiz et al., 2020), particularly urbanisation, as the urban zone was dominated by introduced trees in abundance and species richness. This is also where the greatest accumulation of biogeographic native ranges was found, bearing the risk of facilitating biotic homogenisation at larger scales (Kramer, Bald, et al., 2023).

In São Tomé, 57.7% of the tree species in the urban zone were introduced. Historically, this island being at the intersection of the Portuguese colonial routes may help explain this dominance. In many tropical regions, species introductions are associated with colonialism (Abendroth et al., 2012) and human occupation (Castro et al., 2010). As long‐lived organisms, trees are often living proof of these legacies, such as the many introduced trees around colonial buildings in São Tomé. Other tropical cities do show similar proportions of introduced species (de Souza e Silva et al., 2020), but there is considerable variation, e.g. 75% in Rwanda (Seburanga et al., 2014) and 36.4% in Malawi (Chimaimba et al., 2020).

In the rural zone, over two‐thirds of tree individuals were introduced, which was mostly due to the hyperabundant cocoa trees, but there were also many native species, even though their abundance tended to be lower than in the natural zone. As such, the rural zone could potentially allow for the regeneration of native and endemic species. But it could also facilitate the expansion of introduced species, some of which might become invasive (de Lima et al., 2014), such as trumpet tree (*Cecropia peltata*) or avocado (*Persea americana*) (de Lima et al., 2013), which were present in the natural zone. However, the overall scarcity of introduced species in the natural zone is common to other island forests, for instance in Trinidad (Arnold et al., 2021) and Madagascar (Osen et al., 2021). In fact, native diversity at relatively intact sites may be able to buffer against biological invasions (Delavaux et al., 2023).

### 4.3 How are native biogeographic ranges of tree species distributed across zones?

The natural zone was home to most Afrotropical species, which largely coincided with native species. Nevertheless, Afrotropical species were also well represented in rural and urban zones, in some cases by introduced species (Figure 4). In contrast, most non‐Afrotropical species were associated with the urban zone, reinforcing the fact that this zone is characterised by biotic homogenisation through the introduction of widespread species (Lokatis & Jeschke, 2022). Many of these are useful species, such as the Indomalayan mango tree, which has been introduced across the tropics for its fruits and was the best indicator for the urban zone in São Tomé. The strong historical ties with Brazil facilitated the introduction of many Neotropical species, such as cocoa and coral trees, both of which are indicators of the rural zone. Coral trees are typical shade trees, which grow rapidly and improve the microclimate for cocoa and coffee, the two most important export crops. The top indicator species of both urban and rural zones were breadfruit and jackfruit, which highlights their relevance for both urban and rural dwellers. They were introduced from Oceania and Indomalaya, respectively, for their very large fruits, with breadfruit serving as a staple food on the island.

Endemic abundance and species richness in the natural zone exceeded that of the rural and urban zones. These results contrast with those of temperate cities in Australia (Ives et al., 2016), Greece (Kantsa et al., 2013), and South Africa (Holmes et al., 2012). The scarcity of endemics in São Tomé urban and rural zones might be linked to island species struggling to adapt to anthropogenic environments, especially when they have to compete against numerous introduced species (Sánchez‐Ortiz et al., 2020), that may be planted or self‐propagating. This is not helped by most endemics likely being less valuable to humans, as they tend to have smaller or inedible fruits (Heleno et al., 2022) and take longer to produce timber (de Lima et al., 2013). In addition, people are less familiar with endemics, which are mostly located in the more remote natural zone of this originally uninhabited island, compared to introduced species that arrived with early settlers or to some native species that the first Santomeans may have known from their home countries (de Medeiros et al., 2012). An exception to this is the rural and urban occurrence of *Chytranthus mannii* (Benitez Bosco et al., 2018), an endemic species planted for its edible fruits.

## 5 IMPLICATIONS AND CONCLUSIONS

In São Tomé, the conservation value of urban and rural tree assemblages is very low. They hold few species, most of which are introduced, widespread and not threatened. The natural zone clearly has the highest value for conservation, hosting rich, abundant and diverse tree assemblages that have most of the native, endemic and threatened species. Hence, preserving the natural zone is the most important approach for conserving the island's biodiversity, counteracting biotic homogenisation. However, this could be complemented by species‐specific conservation strategies targeting the few endemic and threatened species that have important populations outside the natural zones, and by exploring approaches to increase the ecological value of rural and urban zones. We therefore propose that conservation strategies on the island should broaden out from the Obô Natural Park for better integration of rural and urban zones into national biodiversity action plans. For instance, natural regeneration can be assisted in the buffer around the Obô Natural Park as well as in other secondary forests, and currently unforested agricultural plots in the rural zone can be turned into agroforests, using native, endemic and threatened tree species. This is already happening as part of “The Restoration Initiative” project in São Tomé and Príncipe, the country's first initiative on Forest and Landscape Restoration. Furthermore, endemics such as *Carapa gogo* or *Chytranthus mannii* may be suitable to diversify tree assemblages of existing shade plantations, enhancing biodiversity and boosting productivity. In addition, urban planting schemes could be initiated that ideally feature climate‐resilient trees of native origin. Creating more awareness about the benefits of protecting endemic species could help make them a symbol of island identity and pride.

Tree diversity was higher in the natural zone, contradicting with the widespread notion that urban zones can harbour high levels of plant diversity. This may partly be due to most studies on urbanisation comparing cities with already highly modified rural hinterlands, such as monoculture fields in industrialised countries, rather than natural ecosystems. Thus, we call for using areas of low human interference as references to assess urban biodiversity, but also for a wider array of metrics that can capture subtle changes in biodiversity. Our results further suggest that the geographic bias of research towards temperate regions may be distorting the current perceptions of how urbanisation influences biodiversity.

## AUTHOR CONTRIBUTIONS

**Lena Strauß:** Conceptualization (lead); formal analysis (lead); investigation (lead); visualization (lead); writing – original draft (lead); writing – review and editing (equal). **Ricardo F. de Lima:** Conceptualization (lead); funding acquisition (lead); investigation (supporting); supervision (lead); writing – review and editing (lead). **Timothy R. Baker:** Conceptualization (lead); supervision (lead); writing – review and editing (lead). **Laura Benitez Bosco:** Conceptualization (equal); investigation (equal); writing – review and editing (equal). **Gilles Dauby:** Conceptualization (equal); investigation (equal); writing – review and editing (equal). **Olivier Lachenaud:** Investigation (equal); writing – review and editing (equal). **Angela Lima:** Investigation (equal); writing – review and editing (supporting). **Dilson Madre Deus:** Investigation (equal); writing – review and editing (supporting). **Maria do Céu Madureira:** Conceptualization (equal); investigation (equal); writing – review and editing (supporting). **Estevão Soares:** Investigation (equal); writing – review and editing (supporting). **Pascoal Sousa:** Investigation (equal); writing – review and editing (supporting). **Tariq Stévart:** Conceptualization (equal); investigation (equal); writing – review and editing (equal). **Martin Dallimer:** Conceptualization (lead); funding acquisition (lead); supervision (lead); writing – review and editing (lead).

## CONFLICT OF INTEREST STATEMENT

The authors declare that they have no competing interests.

## Supporting information


Data S1–S4

## Data Availability

The data that support the findings of this study are available in the supplementary material of this article (Data S1–S4).

## APPENDIX 1

**TABLE A1:** Biogeographic origin and realm (native range) of the 177 tree taxa registered in 81 transects in São Tomé.

Family	Taxon	Origin	Realm	Notes
Anacardiaceae	*Lannea welwitschii*	Native	Afrotropics	All information was extracted from POWO (2023), except for the following markings: * Not listed and assumed as “Introduced” based on native range. + Listed as “Native” or “Native (Endemic)” but might be introduced based on current distribution and on being widely cultivated in the island. ~ Listed as “Introduced” but might be native based on current distribution. ^ Listed as “Native” or not listed but might be a distinct and endemic species. ^F^ Based on Figueiredo et al. (2011). ^T^ Based on ongoing work by the authors. The realm is unknown for taxa that could not be determined to the species level. Species are listed as endemic if their distribution is restricted to the oceanic islands of the Gulf of Guinea. The taxonomy was based on Tropicos (2023). Scientific family or species names not accepted by POWO (2023) are indicated by ^#^.
	*Mangifera indica*	Introduced	Indomalaya^T^	
	*Pseudospondias microcarpa*	Native	Afrotropics	
	*Sorindeia grandifolia*	Native	Afrotropics	
	*Spondias dulcis*	Introduced	Australasia	
	*Spondias mombin*	Introduced	Neotropics	
Anisophylleaceae	*Anisophyllea cabole*	Native	Afrotropics	
Annonaceae	*Annona muricata*	Introduced	Neotropics	
	*Annona squamosa*	Introduced	Neotropics	
	*Cananga odorata*	Introduced	Multiple realms (Indomalaya, Australasia)	
	*Greenwayodendron* aff. *suaveolens*	Native^	Afrotropics	
	*Monodora myristica*	Native	Afrotropics	
	*Xylopia aethiopica*	Native	Afrotropics	
	*Xylopia quintasii*	Native	Afrotropics	
	*Xylopia* sp. nov. Sao Tome^#^	Native (Endemic)^T^	Afrotropics	
Apocynaceae	*Cascabela thevetia*	Introduced	Neotropics	
	*Funtumia africana*	Native	Afrotropics	
	*Funtumia elastica*	Native+	Afrotropics	
	*Rauvolfia caffra*	Native	Afrotropics	
	*Rauvolfia dichotoma*	Native (Endemic)	Afrotropics	
	*Rauvolfia vomitoria*	Native	Afrotropics	
	*Tabernaemontana stenosiphon*	Native (Endemic)	Afrotropics	
Araliaceae	*Astropanax mannii*	Native	Afrotropics	
	*Polyscias quintasii*	Native (Endemic)	Afrotropics	
Asparagaceae	*Dracaena arborea*	Native	Afrotropics	
Asteraceae	*Vernonia amygdalina* ^#^	Native+	Multiple realms (Afrotropics, Neotropics)	
Bignoniaceae	*Crescentia cujete*	Introduced*	Neotropics	
	*Newbouldia laevis*	Native+	Afrotropics	
Burseraceae	*Dacryodes edulis* ^#^	Introduced^T^	Afrotropics	
	*Santiria balsamifera*	Native (Endemic)	Afrotropics	
Cannabaceae	*Celtis gomphophylla*	Native	Afrotropics	
	*Celtis prantlii* ^#^	Native	Afrotropics	
Clusiaceae	*Symphonia globulifera*	Native	Multiple realms (Afrotropics, Neotropics)	
Combretaceae	*Terminalia catappa*	Introduced	Multiple realms (Afrotropics, Indomalaya, Australasia, Oceania)	
Cornaceae	*Alangium* aff. *chinense*	Native^T^	Unknown	
Ebenaceae	*Diospyros ferrea*	Native	Multiple realms (Afrotropics, Indomalaya, Australasia)	
Ehretiaceae^#^	*Ehretia cymosa*	Native	Afrotropics	
Euphorbiaceae	*Anthostema aubryanum*	Native	Afrotropics	
	*Croton stellulifer*	Native (Endemic)	Afrotropics	
	*Discoclaoxylon occidentale*	Native (Endemic)	Afrotropics	
	*Discoglypremna caloneura*	Native	Afrotropics	
	*Euphorbia grandifolia*	Native^T^	Afrotropics	
	*Grossera elongata*	Native (Endemic)	Afrotropics	
	*Hevea brasiliensis*	Introduced	Neotropics	
	*Klaineanthus gabonii* ^#^	Native	Afrotropics	
	*Macaranga monandra*	Native	Afrotropics	
	*Manihot glaziovii* ^#^	Introduced	Neotropics	
	*Pseudagrostistachys africana*	Native	Afrotropics	
	*Shirakiopsis elliptica*	Native	Afrotropics	
	*Tetrorchidium didymostemon*	Native	Afrotropics	
Fabaceae	*Acacia auriculiformis*	Introduced*	Australasia	
	*Albizia chinensis*	Introduced*	Multiple realms (Indomalaya, Australasia)	
	*Albizia falcataria* ^#^	Introduced	Australasia	
	*Albizia lebbeck*	Introduced	Indomalaya	
	*Cassia siamea* ^#^	Introduced	Indomalaya	
	*Cynometra mannii*	Native	Afrotropics	
	*Dialium guineense*	Native+	Afrotropics	
	*Erythrina fusca*	Introduced	Multiple realms (Afrotropics, Neotropics, Indomalaya, Australasia, Oceania)	
	*Erythrina poeppigiana*	Introduced	Neotropics	
	*Erythrina variegata*	Introduced	Multiple realms (Afrotropics, Indomalaya, Australasia, Oceania)	
	*Lonchocarpus sericeus*	Native	Multiple realms (Afrotropics, Neotropics)	
	*Millettia barteri*	Native	Afrotropics	
	*Millettia griffoniana*	Native	Afrotropics	
	*Pentaclethra macrophylla*	Native	Afrotropics	
	*Tamarindus indica*	Introduced	Afrotropics	
Gentianaceae	*Anthocleista scandens*	Native	Afrotropics	
Hypericaceae	*Harungana madagascariensis*	Native	Afrotropics	
Ixonanthaceae	*Phyllocosmus* aff. *sessiliflorus*	Native (Endemic)^T^	Afrotropics	
Lamiaceae	*Gmelina arborea*	Introduced*	Indomalaya	
	*Vitex* sp.	Native^T^	Unknown	
Lauraceae	*Cinnamomum burmannii* ^#^	Introduced	Multiple realms (Indomalaya, Australasia)	
	*Cinnamomum verum*	Introduced	Indomalaya	
	*Persea americana*	Introduced	Neotropics	
Lecythidaceae	*Scytopetalum klaineanum*	Native	Afrotropics	
Malvaceae	*Ceiba pentandra*	Introduced~	Neotropics	
	*Cola acuminata*	Introduced^T^	Afrotropics	
	*Glyphaea brevis*	Native	Afrotropics	
	*Pachira glabra*	Introduced	Neotropics	
	*Sterculia dawei*	Native	Afrotropics	
	*Theobroma cacao*	Introduced	Neotropics	
Melastomataceae	*Memecylon myrianthum*	Native^T^	Afrotropics	
Meliaceae	*Carapa gogo*	Native (Endemic)	Afrotropics	
	*Cedrela odorata*	Introduced	Neotropics	
	*Trichilia grandifolia*	Native (Endemic)	Afrotropics	
Moraceae	*Artocarpus altilis*	Introduced	Oceania	
	*Artocarpus camansi*	Introduced*	Australasia	
	*Artocarpus heterophyllus*	Introduced	Indomalaya	
	*Castilla elastica*	Introduced	Neotropics	
	*Ficus chlamydocarpa*	Native	Afrotropics	
	*Ficus exasperata*	Native	Multiple realms (Afrotropics, Indomalaya)	
	*Ficus mucuso*	Native	Afrotropics	
	*Ficus sur*	Native	Afrotropics	
	*Ficus thonningii*	Native	Afrotropics	
	*Mesogyne insignis*	Native^	Afrotropics	
	*Milicia excelsa*	Native	Afrotropics	
	*Treculia africana*	Native+	Afrotropics	
	*Trilepisium madagascariense*	Native	Afrotropics	
Moringaceae	*Moringa oleifera*	Introduced*	Indomalaya	
Myristicaceae	*Pycnanthus angolensis*	Native	Afrotropics	
	*Staudtia pterocarpa*	Native (Endemic)	Afrotropics	
Myrtaceae	*Eucalyptus torelliana* ^#^	Introduced*	Australasia	
	*Eugenia brasiliensis*	Introduced	Neotropics	
	*Eugenia uniflora*	Introduced	Neotropics	
	*Psidium guajava*	Introduced	Neotropics	
	*Syzygium guineense*	Native^T^^	Afrotropics	
Ochnaceae	*Campylospermum reticulatum*	Native	Afrotropics	
	*Campylospermum vogelii*	Native	Afrotropics	
	*Idertia axillaris*	Native	Afrotropics	
	*Rhabdophyllum arnoldianum*	Native	Afrotropics	
	*Rhabdophyllum calophyllum*	Native	Afrotropics	
Olacaceae	*Heisteria parvifolia*	Native	Afrotropics	
	*Strombosia grandifolia*	Native	Afrotropics	
	*Strombosia* sp. nov. Sao Tome^#^	Native (Endemic)^T^	Afrotropics	
Oleaceae	*Olea capensis*	Native	Afrotropics	
Oxalidaceae	*Averrhoa bilimbi*	Introduced*	Australasia	
Phyllanthaceae	*Amanoa* cf. *bracteosa*	Native^T^	Afrotropics^T^	
	*Antidesma vogelianum*	Native	Afrotropics	
	*Bridelia micrantha*	Native	Afrotropics	
	*Cleistanthus libericus*	Native	Afrotropics	
	*Maesobotrya glabrata*	Native (Endemic)	Afrotropics	
	*Margaritaria discoidea*	Native	Afrotropics	
	*Protomegabaria stapfiana*	Native	Afrotropics	
	*Thecacoris manniana* ^#^	Native (Endemic)^F^	Afrotropics	
	*Uapaca vanhouttei*	Native^T^	Afrotropics	
Primulaceae	*Rapanea melanophloeos* ^#^	Native	Afrotropics	
Proteaceae	*Grevillea robusta*	Introduced	Australasia	
Putranjivaceae	*Drypetes glabra*	Native (Endemic)	Afrotropics	
	*Drypetes henriquesii*	Native (Endemic)	Afrotropics	
	*Drypetes principum*	Native	Afrotropics	
Rhamnaceae	*Lasiodiscus rozeirae*	Native (Endemic)	Afrotropics	
	*Maesopsis eminii*	Native	Afrotropics	
	*Ziziphus abyssinica*	Native+	Afrotropics	
Rhizophoraceae	*Cassipourea gummiflua*	Native	Afrotropics	
Rubiaceae	*Aidia quintasii*	Native (Endemic)	Afrotropics	
	*Aulacocalyx pallens*	Native	Afrotropics	
	*Belonophora coffeoides*	Native	Afrotropics	
	*Bertiera racemosa*	Native	Afrotropics	
	*Cinchona pubescens* ^#^	Introduced	Neotropics	
	*Coffea canephora*	Introduced^F^	Afrotropics	
	*Craterispermum cerinanthum*	Native	Afrotropics	
	*Hymenodictyon biafranum*	Native	Afrotropics	
	*Morinda lucida*	Native	Afrotropics	
	*Oxyanthus speciosus*	Native	Afrotropics	
	*Pauridiantha floribunda*	Native	Afrotropics	
	*Pauridiantha insularis*	Native (Endemic)	Afrotropics	
	*Pavetta monticola*	Native (Endemic)	Afrotropics	
	*Pouchetia* aff. *parviflora*	Native (Endemic)^T^	Afrotropics	
	*Psychotria grumilea* ^#^	Native (Endemic)	Afrotropics	
	*Psychotria venosa*	Native	Afrotropics	
	*Psydrax sanguinolenta* sp. nov.^#^	Native^T^	Afrotropics^T^	
	*Psydrax subcordata* ^#^	Native	Afrotropics	
	*Rothmannia urcelliformis*	Native	Afrotropics	
	*Tarenna nitiduloides*	Native (Endemic)	Afrotropics	
Rutaceae	*Citrus* × *aurantium*	Introduced	Hybrid	
	*Zanthoxylum gilletii*	Native	Afrotropics	
	*Zanthoxylum thomense*	Native	Afrotropics	
Salicaceae	*Casearia barteri*	Native	Afrotropics	
	*Homalium henriquesii*	Native	Afrotropics	
	*Ophiobotrys zenkeri*	Native	Afrotropics	
Sapindaceae	*Allophylus africanus*	Native	Afrotropics	
	*Allophylus grandifolius*	Native	Afrotropics	
	*Blighia sapida*	Native+	Afrotropics	
	*Chytranthus mannii*	Native (Endemic)+	Afrotropics	
Sapotaceae	*Gambeya africana*	Native	Afrotropics	
	*Gambeya albida*	Native+	Afrotropics	
	*Manilkara obovata*	Native	Afrotropics	
	*Synsepalum revolutum*	Native	Afrotropics	
	*Synsepalum* sp. nov. 1 Sao Tome^#^	Native (Endemic)^T^	Afrotropics	
	*Synsepalum* sp. nov. 2 Sao Tome^#^	Native (Endemic)^T^	Afrotropics	
Simaroubaceae	*Hannoa klaineana* ^#^	Native	Afrotropics	
Solanaceae	*Cestrum laevigatum*	Introduced	Neotropics	
Stilbaceae	*Nuxia congesta*	Native	Afrotropics	
Thymelaeaceae	*Dicranolepis thomensis*	Native (Endemic)	Afrotropics	
	*Peddiea thomensis*	Native (Endemic)	Afrotropics	
Urticaceae	*Cecropia peltata*	Introduced	Neotropics	
Violaceae	*Rinorea chevalieri*	Native (Endemic)	Afrotropics	
Vitaceae	*Leea tinctoria*	Native (Endemic)	Afrotropics	

**TABLE A2:** Significant indicator values (significance levels: **p* ≤ .05, ***p* ≤ .01) of tree taxa for the urban, rural and natural zones, as well as for combinations of them.

Family	Taxon	Indicator value	Significance level
*Urban*			
Anacardiaceae	*Mangifera indica*	0.810	**
Bignoniaceae	*Newbouldia laevis*	0.750	**
Annonaceae	*Annona muricata*	0.616	**
Combretaceae	*Terminalia catappa*	0.577	**
Anacardiaceae	*Spondias mombin*	0.528	*
Sapotaceae	*Gambeya albida*	0.516	**
Lamiaceae	*Gmelina arborea*	0.447	**
*Rural*			
Malvaceae	*Theobroma cacao*	0.920	**
Fabaceae	*Erythrina poeppigiana*	0.775	**
Euphorbiaceae	*Tetrorchidium didymostemon*	0.708	**
Urticaceae	*Cecropia peltata*	0.635	**
Anacardiaceae	*Pseudospondias microcarpa*	0.543	**
Cannabaceae	*Celtis gomphophylla*	0.533	**
Euphorbiaceae	*Macaranga monandra*	0.440	*
Fabaceae	*Albizia lebbeck*	0.420	**
*Natural*			
Salicaceae	*Homalium henriquesii*	0.803	**
Salicaceae	*Casearia barteri*	0.778	**
Rubiaceae	*Pauridiantha floribunda*	0.724	**
Phyllanthaceae	*Antidesma vogelianum*	0.680	**
Lecythidaceae	*Scytopetalum klaineanum*	0.672	**
Phyllanthaceae	*Thecacoris manniana*	0.672	**
Rubiaceae	*Oxyanthus speciosus*	0.657	**
Sapotaceae	*Gambeya africana*	0.648	**
Burseraceae	*Santiria balsamifera*	0.622	**
Annonaceae	*Xylopia* sp. nov. Sao Tome	0.599	**
Rubiaceae	*Craterispermum cerinanthum*	0.596	**
Myrtaceae	*Syzygium guineense*	0.596	**
Phyllanthaceae	*Uapaca vanhouttei*	0.596	**
Phyllanthaceae	*Cleistanthus libericus*	0.587	**
Putranjivaceae	*Drypetes henriquesii*	0.568	**
Annonaceae	*Greenwayodendron* aff. *suaveolens*	0.568	**
Euphorbiaceae	*Grossera elongata*	0.568	**
Olacaceae	*Heisteria parvifolia*	0.539	**
Phyllanthaceae	*Protomegabaria stapfiana*	0.539	**
Clusiaceae	*Symphonia globulifera*	0.539	**
Rubiaceae	*Psychotria venosa*	0.532	**
Ochnaceae	*Rhabdophyllum arnoldianum*	0.525	**
Rhizophoraceae	*Cassipourea gummiflua*	0.520	**
Rubiaceae	*Aulacocalyx pallens*	0.508	**
Euphorbiaceae	*Croton stellulifer*	0.508	**
Sapotaceae	*Synsepalum revolutum*	0.508	*
Euphorbiaceae	*Klaineanthus gabonii*	0.506	**
Phyllanthaceae	*Amanoa* cf. *bracteosa*	0.475	**
Rubiaceae	*Psydrax sanguinolenta* sp. nov.	0.475	**
Apocynaceae	*Rauvolfia dichotoma*	0.475	**
Myristicaceae	*Staudtia pterocarpa*	0.475	*
Rubiaceae	*Aidia quintasii*	0.440	**
Sapindaceae	*Blighia sapida*	0.440	**
Phyllanthaceae	*Maesobotrya glabrata*	0.440	**
Apocynaceae	*Tabernaemontana stenosiphon*	0.440	*
Simaroubaceae	*Hannoa klaineana*	0.424	*
Sapotaceae	*Synsepalum* sp. nov. 1 Sao Tome	0.421	*
Putranjivaceae	*Drypetes glabra*	0.402	*
Rubiaceae	*Pavetta monticola*	0.402	*
Olacaceae	*Strombosia* sp. nov. Sao Tome	0.402	*
Thymelaeaceae	*Dicranolepis thomensis*	0.359	*
Euphorbiaceae	*Discoclaoxylon occidentale*	0.359	*
Euphorbiaceae	*Discoglypremna caloneura*	0.359	*
Euphorbiaceae	*Pseudagrostistachys africana*	0.359	*
*Urban and rural*			
Moraceae	*Artocarpus altilis*	0.854	**
Moraceae	*Artocarpus heterophyllus*	0.775	**
Meliaceae	*Cedrela odorata*	0.705	**
Rubiaceae	*Morinda lucida*	0.663	**
Moraceae	*Milicia excelsa*	0.663	**
Moraceae	*Ficus exasperata*	0.648	**
Burseraceae	*Dacryodes edulis*	0.583	**
Myrtaceae	*Psidium guajava*	0.469	*
Moraceae	*Castilla elastica*	0.444	*
*Urban and natural*			
Asparagaceae	*Dracaena arborea*	0.496	*
*Rural and natural*			
Myristicaceae	*Pycnanthus angolensis*	0.737	**
Apocynaceae	*Funtumia africana*	0.534	**

**TABLE A3:** Hierarchical partitioning, combining unique and common fractions, of environmental variables: Altitude, precipitation, remoteness, slope, topography, cloud cover.

	Individual effect (%)
Altitude	20.52
Precipitation	24.19
Remoteness	17.07
Slope	9.61
Topography	14.41
Cloud cover	14.10
**Total (%)**	**100.00**

**TABLE A4:** Variation partitioning between environment (altitude, precipitation, remoteness, slope, topography, cloud cover), zone (urban, rural, natural), and PCNM (principal coordinates of neighbour matrices) explaining variation (*R*‐squared) of tree assemblages.

		Fraction of explained variation (%)
Unique to	Environment	3.66
	Zone	1.88
	PCNM	8.41
Common to	Environment and zone	1.96
	Environment and PCNM	2.71
	Zone and PCNM	6.15
	Environment, zone, and PCNM	14.56
**Total explained variation (%)**		**39.32**
