# Supplementary material for: Widespread introduced species dominate the urban tree assemblage on the endemic‐rich tropical island of São Tomé
Source: Ecol Evol. 2024 Sep 1;14(9):e70153. doi: 10.1002/ece3.70153 (PMC11366497; doi:10.1002/ece3.70153)
Supplement: Supplementary file 1 — Data S1–S4 [file ECE3-14-e70153-s001.zip › ECE3_70153_Legends.docx]

*Dataset S1: Community data of the 177 tree taxa registered in 81 transects in São Tomé, including zone (urban, rural, natural).*

*Dataset S2: Biogeographic origin and realm (native range) of the 177 tree taxa registered in 81 transects in São Tomé (based on Figueiredo et al., 2011; POWO, 2023).*

*Dataset S3: Environmental variables for 81 transects in São Tomé (continuous data standardised): alt = altitude, pre = precipitation, rem = remoteness, slo = slope, top = topography including flat areas (1), valleys (2), middle slopes (3), upper slopes (4), ridges (5) (Soares et al., 2020); clo = cloud cover (Wilson and Jetz, 2016).*

*Code S4: R script for Figures 2-4 and permutation tests.*
